# Correspondence

**Published:** 1903-11

**Authors:** G. Frank Lydston

**Affiliations:** Chicago


### CORRESPONDENCE.

CHICAGO, October 27, 1903.

*To the Members of the Georgia State Medical Society:*

GENTLEMEN :—At the last meeting of your association a paper was read by Dr. W. E. Fitch, of Savannah, Ga., on "The Remote Effects of Latent Gonorrhea in the Female." This was published in the *Annals of Gynecology and Pediatry* for September, 1903. I presume that the members of the society accepted the paper as original. It was, however, taken bodily—the greater part of it—from a paper published by me in a collection of essays and addresses in 1892, my monograph on "Gonorrhea" (Davis's Leisure Library) in 1892, and my text-book on Genito-Urinary Diseases, published in 1899. A comparison of Dr. Fitch's paper with my text-book, page 163, is alone sufficient. The copy is almost verbatim. Aside from the case Dr. Fitch reported, not a paragraph of the article could be considered original.

I write this because I feel that your society as well as I have been imposed upon. G. FRANK LYDSTON, M.D.

---

IN his annual report as commander of the department of Texas, General Frederick D. Grant renews the argument for restoration of the post exchange or canteen. The canteen, he says, is the enlisted soldier's club-room, into which no vice or degradation can intrude. There the soldier is guaranteed certain privileges which, so long as not abused, prove a bulwark against the temptations and dangers that now surround almost every post in the army, and especially border posts. To close the doors of the soldier's garrison club and send him out into the haunts of iniquity and vice run by moral vultures is a wrong to the soldier as well as to the community in which the soldier is stationed.—*Exchange.*

